# Total ischemic myocardium is a powerful predictor for adverse cardiac events

**DOI:** 10.1186/1532-429X-17-S1-P95

**Published:** 2015-02-03

**Authors:** Dominik Buckert, Nils Dyckmanns, Volker Rasche, Wolfgang Rottbauer, Peter Bernhardt

**Affiliations:** Innere Medizin II, Uniklinik Ulm, Ulm, Germany

## Background

Imaging techniques may contribute to the proper risk assessment of patients with coronary artery disease (CAD). Especially, cardiac magnetic resonance imaging (CMR) is able to provide important prognostic information by detection of myocardial scarring (late gadolinium enhancement, LGE) and reversible ischemia. Therefore, objective of the present study was to quantify the extent of total ischemic myocardium (TIM) by the combination of LGE and reversible ischemia by CMR and to correlate the results with long-term outcome and prognosis in patients with known or suspected CAD.

## Methods

Consecutive patients referred for CMR imaging were prospectively screened and enrolled from 2003 to 2007. Besides functional and volumetric assessment, adenosine perfusion and LGE imaging were performed on a 1.5 T whole-body scanner. TIM was assessed and quantified by combination of reversible ischemia and LGE as follows: perfusion deficits and LGE were identified and quantified by a semi-automatic approach on short-axis images. TIM was defined as area of reversible perfusion deficit plus area of LGE and expressed as percentage of global myocardium. Primary endpoint was defined as cardiac death, nonfatal myocardial infarction or stroke.

## Results

Our study population consisted of 845 consecutive patients. Mean follow-up was 4.1 ± 1.8 years. During this time, 61 primary endpoints occurred with total yearly event rates of 2.1% in the first, 1.2% in the second and .7% in the third year, respectively. A 36% cutoff for TIM was defined by ROC analysis. Primary endpoints occurred in 15 patients with TIM higher than the cutoff (event rate 33%). In contrast, primary endpoints were observed in only 5.8% (46) of patients with low or absent TIM (N=15 [33%] vs. N=46 [5.8%], p<.001). On multivariate analysis, TIM was the strongest independent predictor for the occurrence of a primary endpoint with a hazard ratio of 3.4.

## Conclusions

TIM can easily be assessed and quantified by the provided CMR algorithm in daily clinical routine. TIM >36% of myocardial mass is strongly associated with the occurrence of major clinical adverse events and thus owns important prognostic value.

## Funding

None.

